# Are park proximity and park features related to park use and park-based physical activity among adults? Variations by multiple socio-demographic characteristics

**DOI:** 10.1186/s12966-014-0146-4

**Published:** 2014-12-06

**Authors:** Andrew T Kaczynski, Gina M Besenyi, Sonja A Wilhelm Stanis, Mohammad Javad Koohsari, Katherine B Oestman, Ryan Bergstrom, Luke R Potwarka, Rodrigo S Reis

**Affiliations:** Department of Health Promotion Education and Behavior, Arnold School of Public Health, University of South Carolina, 915 Greene Street, Room 529, Columbia, SC 29201 USA; Prevention Research Center, Arnold School of Public Health, University of South Carolina, Columbia, SC USA; Department of Parks, Recreation, and Tourism, University of Missouri-Columbia, 105 ABNR Building, Columbia, MO USA; Behavioural Epidemiology Laboratory, Baker IDI Heart and Diabetes Institute, Melbourne, Australia; McCaughey VicHealth Centre for Community Wellbeing, Melbourne School of Population and Global Health, University of Melbourne, Melbourne, Australia; Riley County Health Department, 2030 Tecumseh Road, Manhattan, KS USA; Department of Geography, 800 West College Ave, Gustavus Adolphus College, St. Peter, MN USA; Department of Recreation and Leisure Studies, University of Waterloo, Waterloo, Ontario Canada; School of Health and Biosciences, Pontifica Universidade Catolica do Parana, Curitiba, Brazil

**Keywords:** Parks, Physical activity, Proximity, Facilities, Urban design, Built environment, Neighborhood

## Abstract

**Background:**

Parks are valuable resources for physical activity (PA) given their widespread availability and low cost to maintain and use. Both proximity to parks and the availability of particular features are important correlates of PA. However, few studies have explored multiple measures of proximity simultaneously or the specific facilities associated with park use and park-based PA among adults, let alone differences across socio-demographic characteristics. The purpose of this study was to examine associations between park proximity and park facilities and adults’ park use and park-based PA, while also exploring differences by gender, age, race, and income.

**Methods:**

Data on monthly park use and weekly amount of PA undertaken in parks were collected via a mail survey of adults from randomly-selected households (n = 893) in Kansas City, Missouri (KCMO) in 2010–2011. Three measures of park proximity were calculated within 1 mile of participating households: distance to the closest park, number of parks, and total park area. All parks in KCMO were audited using the Community Park Audit Tool to determine the availability of 14 park facilities within 1 mile of each participant (e.g., trail, playground, tennis court). Multilevel logistic regression was used to examine the relationship between each of park use and park-based PA and 1) three measures of park proximity, and 2) the availability of 14 park facilities within 1 mile of participants. Separate analyses were conducted by gender, age, race, and income, while controlling for all socio-demographic characteristics and BMI.

**Results:**

Across all sub-samples, distance to the closest park was not significantly related to either park use or park-based PA. However, numerous significant associations were found for the relationship of number of parks and amount of park space within 1 mile with both outcomes. As well, diverse facilities were associated with park use and park-based PA. For both park proximity and facilities, the significant relationships varied widely across gender, age, race, and income groups.

**Conclusions:**

Both park proximity and park facilities are related to park use and park-based PA. Understanding how such associations vary across demographic groups is important in planning for activity-friendly parks that are responsive to the needs of neighborhood residents.

## Background

Engaging in regular physical activity (PA) confers substantial health benefits throughout all life stages and across demographic groups [1-3], including a reduced risk of obesity and chronic diseases such as type 2 diabetes, cardiovascular disease, and some cancers [4,5]. Nevertheless, population-wide PA participation has declined over the last few decades across the world [6]. For example, across 122 countries, approximately one-third of adults are physically inactive, ranging from 17% in southeast Asia to about 43% in the Americas and the eastern Mediterranean [7].

Given the limited success of individually-based approaches to boost healthy behaviors such as PA, ecological models are gaining popularity in both research and practice [8,9]. In particular, ecological models emphasize the importance of urban design policy and environmental factors for both facilitating and inhibiting community health [10]. The built environment can be defined as “the human-made space in which people live, work, and recreate on a day-to-day basis” [11] and parks are one key resource in communities for encouraging PA and reducing obesity among residents of all ages [12-14]. Parks can enhance the active ambience of the overall neighborhood environment and they can also be destinations for walking to as well as settings for a wide variety of recreational activities [15-19].

A growing body of literature has examined how different aspects of parks are associated with PA participation, including factors such as proximity, design features, and elements of the surrounding environment [20-27]. However, this paper aims to address at least four limitations within research on parks and adults’ PA to date. First, many studies have examined the role of proximity to parks and adult PA, including some which have looked at aggregate amounts of green space in participants’ neighborhoods [28,29]. However, most research on park proximity and PA has considered only the shortest distance to the nearest park, which ignores the possibility of having several parks (and of different sizes) within a neighborhood that may influence residents’ behaviors. For example, one recent study of public open spaces (POSs) in Melbourne reported that other proximity measures such as the number of POSs and the total area of POSs within 1 km were also associated with POS-related PA [30].

Second, most research has employed context-free outcomes such as total PA (derived via self-report or electronic monitoring) when examining relationships between parks and PA [31-33]. It has been noted that such global outcomes potentially include a substantial amount of PA which is unrelated to parks (e.g., walking on neighborhood streets for shopping), which could bias the observed relationships [34]. Only a limited number of studies have used context-specific measures of park-based PA or active travel to parks in considering how proximity to or specific features of parks influence PA [22,23,30]. For example, Schipperijn et al. [25] found associations between characteristics of urban green space (e.g., size and features) and PA in the nearest urban green space, while no associations were found with general PA.

Third, few studies among adults have comprehensively examined how the specific features of parks are associated with park-based PA. Kaczynski et al. [23] reported that park features were more important than factors such as size and distance in determining use by residents for PA. Likewise, Sugiyama et al. [26] found that the presence of a large attractive park within walking distance of someone’s home may be more important in encouraging adults’ sufficient walking than having a less desirable park within a shorter distance. However, despite a few notable exceptions [25,35], the majority of research on park features and PA participation to date has been conducted among youth populations [36-40].

Finally, rarely have researchers considered how the relationship between park proximity and features and adults’ PA may vary according to factors such as gender, age, income, or race/ethnicity. Numerous studies report that overall PA participation and park use patterns differ across socio-demographic groups and that preferences for specific park features and attributes are also heterogeneous [41-46]. However, most research on parks and PA has failed to consider potential differences according to residents’ socio-demographic characteristics. As an example, Kaczynski et al. [16] found that living near more parks and more parkland were more positively associated with PA among women than men and among younger (18–34 years) and older (55+ years) adults than middle-aged (35–54 years) adults. Better understanding the influence of park attributes on park-related PA across different population groups can aid in (re)designing parks and open spaces within neighborhoods to create more activity-friendly built environments for all.

### Purpose

The purpose of the present study was to understand how multiple measures of proximity to parks and specific park features were associated with park-based PA among adults and how such relationships may vary according to several socio-demographic characteristics.

## Methods

### Study setting and sample

This study took place in 2010 and was part of the Kansas City Parks and Physical Activity Project, a multi-stage investigation of the influence of park and neighborhood characteristics on youth and adult PA and obesity in Kansas City, Missouri (KCMO). The study procedures were approved by the Institutional Review Boards at Kansas State University and the University of Missouri. KCMO has a population of almost one half million (459,787) residents from diverse racial/ethnic and socioeconomic backgrounds and contains 219 parks ranging in size from 0.16 to 1805 acres and possessing a diverse array of facilities and amenities.

With the assistance of a market research company (Survey Sampling International, Shelton, CT), an initial random sample of almost 4000 residential addresses (n = 3906) was identified that were geographically-dispersed across KCMO. This was accomplished using an initial set of 60 diverse parks that were part of a broader study and then by identifying all census blocks within 0.5 miles of those parks. From those census blocks, we then selected a random sample of 66 addresses around each park. A self-administered questionnaire was sent to sample households using a modified Dillman [47] mailing protocol. It was requested that a single adult living within the household complete the survey. From October through December 2010, the mailing protocol included an initial cover letter and lengthy questionnaire, a thank you/reminder postcard, and three additional mailings of follow-up questionnaires to those who had not yet responded. Of the 3906 questionnaires that were mailed out, 649 were returned by the postal service as undeliverable and 893 were returned completed. This resulted in a 27.4% response rate, which is comparable to other similar studies about parks within the general population [48,49]. Compared to the broader KCMO adult population, our sample was slightly more female and older with a greater proportion of Whites respondents, but with similar income levels.

### Measures

#### Park proximity

All parks in the City of KCMO directory (n = 219) were visited to determine if they were present, accessible, and useable for recreation (n = 146). In this study, we examined three measures of park proximity objectively using Geographic Information Systems (GIS) – distance to the closest park, total number of parks, and total park acreage. The distance from the participant’s address to the geometric centroid of the nearest park was calculated using the street network distance, which has been shown to be more appropriate in simulating walking behavior than Euclidean distance [50,51]. A variable was created to indicate whether each participant had their closest park within less than ½ mile, between ½ mile to 1 mile, or farther than one mile. In addition, a 1 mile network buffer was created around each participant’s address to determine the total number of parks and the total park acreage that fell into this catchment area, with a park or its acreage included if the park’s centroid fell within this distance. Although no common standard exists for proximity to parks, this 1 mile-buffer was thought to be a reasonable walking distance and is similar to that used in several past studies of PA and built environment attributes [31,52,53].

#### Park facilities

All parks within 1 mile of any participating household (n = 146) were assessed by two trained auditors using the Community Park Audit Tool (CPAT). The CPAT has demonstrated excellent inter-rater reliability for the vast majority of its items [54] and park facilities were defined as areas in the park that could be used for PA [23]. Specifically, the CPAT provided in-depth information regarding the presence/absence of 14 park facilities, including playgrounds, sports fields (e.g., football, soccer), baseball fields, swimming pools, splash pads, basketball courts, tennis courts, volleyball courts, trails, fitness stations, skate parks, dog parks, green spaces, and lakes.

#### Park use and park-based PA

Self-reported park use was captured with a question from the Physical Activity in Park Settings (PA-PS) questionnaire, which has demonstrated reliability [55]. Specifically, participants reported whether they had visited a park within the past 30 days (yes/no). A park was defined for respondents as “a public park or outdoor recreation area in the community that is designed for active or passive use” [56]. The second outcome variable asked about the amount of time (minutes) spent being physically active in a park in a usual week. Because of the large proportion of people who reported not having used a park in the last 30 days (56.3%) or who reported zero minutes of PA in a park in a usual week (55.1%), both park use and park-based PA were dichotomized as ‘some’ versus ‘none’.

#### Participant characteristics

Finally, participant demographics captured in the study questionnaire included gender, income, race, age, and height and weight.

### Analyses

Descriptive statistics were used to characterize the study participants’ socio-demographic characteristics and park use and park-based PA behaviors. Multilevel logistic regression analyses (with census tract of residence as the level 2 variable) were used to examine the relationship between each outcome variable (park use and park PA) and 1) three measures of park proximity (closest park, number of parks, park acreage), and 2) the availability of 14 park facilities within 1 mile. In addition to examining these associations among the full sample of participants, separate analyses were conducted by gender (male/female), income (<$25,000, $25,000-74,999, $75,000+), race (White, Black), and age (18–39, 40–59, 60+ years), while controlling for all socio-demographic characteristics and body mass index calculated from height and weight. All analyses were conducted using SPSS 19.0 and findings were considered significant at p < .05.

## Results

### Sample characteristics

Table 1 shows the characteristics of the study sample. Almost two-thirds (60.8%) of participants were female, the mean age was 50.9 years (SD = 16.5; range = 18 to 98), half (49.7%) were from middle-income households ($25,000-74,999), two-thirds were White (66.9%), and more than half were either overweight (36.0%) or obese (25.1%). Almost half of participants had used parks within the past month (43.7%), while a similar number reported engaging in some park-based physical activity in a usual week (44.9%). Table 2 also provides descriptive statistics for the full sample for all park proximity and park feature variables examined in this study.

**Table 1:** **Sample characteristics**

**Participant characteristic**	**%**
Gender	
Female	60.8%
Male	39.2%
Annual Household Income	
Less than $25,000	24.8%
$25,000-74,999	49.7%
$75,000 or more	25.5%
Race/Ethnicity	
White, non-Hispanic	66.9%
Black, non-Hispanic	24.5%
Hispanic/Latino of any race	4.7%
Other, non-Hispanic	2.3%
Asian, non-Hispanic	1.6%
Age	
18-39 years	29.6%
40-59 years	24.3%
60 years or more)	31.3%
Body Mass Index	
Underweight or Normal Weight	38.9%
Overweight	36.0%
Obese	25.1%
Park Use Within Past Month	
Yes	43.7%
No	56.3%
Park-Based Physical Activity in Usual Week	
Yes	44.9%
No	55.1%

**Table 2:** **Park proximity and facilities descriptive statistics**

**Park variable**	**%**
Closest Park	
Less than ½ mile	44.3%
½ to 1 mile	40.6%
More than 1 mile	15.0%
Number of Parks within 1 mile	
0 parks	15.0%
1 park	28.4%
2 parks	21.6%
3 parks	34.7%
Park Space	
0 acres	15.0%
0.1-19.9 acres	32.0%
20-49.9 acres	28.2%
50 or more acres	24.5%
Park Facilities	
Playground	73.2%
Sports Field	22.7%
Baseball Field	55.0%
Swimming Pool	13.0%
Splash Pad	15.8%
Basketball Court	37.4%
Tennis Court	33.3%
Volleyball Court	4.2%
Trail	62.7%
Fitness Station	5.6%
Skate Park	7.6%
Dog Park	2.7%
Green Space	84.5%
Lake	21.5%

Percentages for park facilities indicate the proportion of the sample with each facility within a park within 1 mile from home.

### Association of park proximity with park use and park-based PA

As shown in the top half of Tables 3 and 4, for all sub-samples, distance to the closest park was not statistically significantly related to both park use and park-based PA. However, several statistically significant associations (p < .05) were found for the relationship of number of parks and amount of park space within 1 mile with both outcomes among several participant groups. For example, White participants with two or three or more parks and the full sample, females, and high income participants with three or more parks were significantly more likely to report having used a park in the past 30 days than those with no parks within 1 mile; perhaps not surprisingly, these results suggest that as the number of parks increased, so too did the odds of having used a park (Table 3). As well, with at least 20 acres of park space within 1 mile, there were significantly greater odds of park use for the full sample, females, low income participants, and both White and Black participants compared to those with no park space nearby (Table 3).

**Table 3:** **Association of park proximity and park facilities with park use by socio-demographic characteristics**

**Park variable**	**Full sample**		**Gender**				**Income**						**Race**				**Age**					
			**Males**		**Females**		**Low income**		**Medium income**		**High income**		**White**		**Black**		**18-39 years**		**40-59 years**		**60+ years**	
	**OR**	**95% CI**	**OR**	**95% CI**	**OR**	**95% CI**	**OR**	**95% CI**	**OR**	**95% CI**	**OR**	**95% CI**	**OR**	**95% CI**	**OR**	**95% CI**	**OR**	**95% CI**	**OR**	**95% CI**	**OR**	**95% CI**
Closest park																						
Less than ½ mile	1.00		1.00		1.00		1.00		1.00		1.00		1.00		1.00		1.00		1.00		1.00	
½ mile to 1 mile	0.60	0.41-0.87	0.84	0.45-1.58	0.49	0.30-0.80	0.42	0.19-0.94	0.83	0.49-1.42	0.37	0.16-0.85	0.63	0.40-1.01	0.43	0.18-1.05	0.40	0.19-0.81	0.88	0.478-1.63	0.46	0.21-0.99
More than 1 mile	0.54	0.31-0.93	0.73	0.29-1.88	0.48	0.23-0.98	0.16	0.03-0.85	0.92	0.43-1.96	0.38	0.12-1.18	0.51	0.27-0.95	0.12	0.01-2.36	0.60	0.21-0.81	0.389	0.15-1.00	0.75	0.28-2.03
Number of parks																						
0 parks	1.00		1.00		1.00		1.00		1.00		1.00		1.00		1.00		1.00		1.00		1.00	
1 park	0.82	0.45-1.46	0.74	0.29-1.90	0.82	0.38-1.76	2.59	0.46-14.48	0.73	0.33-1.59	0.45	0.14-1.49	0.69	0.36-1.40	5.58	0.29-105.85	0.54	0.18-1.63	1.36	0.51-3.60	0.54	0.19-1.54
2 parks	1.56	0.86-2.86	1.41	0.51-3.91	1.54	0.70-3.36	4.52	0.77-26.66	0.85	0.37-1.92	3.55	0.94-13.49	**2.11**	**1.07-4.18**	2.68	0.13-57.14	0.95	0.32-2.82	2.48	0.90-6.80	1.53	0.50-4.76
3 or more parks	**2.31**	**1.30-4.10**	2.10	0.80-5.49	**2.32**	**1.09-4.95**	5.53	0.98-31.30	1.46	0.67-3.20	**5.03**	**1.40-18.09**	**3.13**	**1.58-6.22**	8.01	0.43-149.26	1.96	0.70-5.53	5.12	1.89-13.91	1.20	0.41-3.54
Park space																						
0 acres	1.00		1.00		1.00		1.00		1.00		1.00		1.00		1.00		1.00		1.00		1.00	
0.1-19.9 acres	1.10	0.63-1.93	0.86	0.34-2.17	1.19	0.56-2.51	6.76	0.61-74.50	0.95	0.44-2.04	0.67	0.21-2.14	1.00	0.52-1.92	3.56	0.21-60.64	0.75	0.25-2.21	1.60	0.61-4.19	0.72	0.26-2.06
20-49.9 acres	**2.18**	**1.22-3.87**	1.57	0.58-4.26	**2.63**	**1.23-5.65**	**14.70**	**1.33-161.92**	1.39	0.64-3.01	4.00	0.95-16.87	**2.81**	**1.42-5.56**	5.61	0.32-97.83	1.81	0.66-4.99	2.27	0.84-6.17	1.92	0.67-5.51
50 or more acres	**2.47**	**1.38-4.44**	1.70	0.64-4.55	**2.84**	**1.31-6.17**	**35.39**	**3.24-386.61**	0.99	0.44-2.22	3.22	0.87-11.86	**2.54**	**1.26-5.10**	**17.46**	**1.07-286.00**	1.72	0.61-4.87	5.29	1.81-15.45	1.14	0.36-3.55
Park facilities^1^																						
Playground	**1.98**	**1.32-2.97**	1.52	0.77-2.97	**2.30**	**1.36-3.90**	**2.90**	**1.07-7.88**	1.43	0.82-2.47	**3.35**	**1.34-8.34**	**2.34**	**1.44-3.81**	**3.19**	**1.01-10.14**	**2.37**	**1.13-4.97**	2.25	0.95-5.35	1.48	0.68-3.23
Sports Field	1.05	0.70-1.58	0.72	0.37-1.39	1.23	0.71-2.13	0.85	0.38-1.87	1.24	0.68-2.25	0.96	0.37-2.45	1.49	0.90-2.48	0.82	0.26-2.57	1.37	0.61-3.07	1.98	0.90-4.37	0.64	0.26-1.61
Baseball field	**1.73**	**1.20-2.50**	1.27	0.70-2.30	**2.01**	**1.22-3.30**	**5.31**	**1.79-15.78**	1.11	0.68-1.82	**2.36**	**1.08-5.14**	**1.95**	**1.26-3.02**	**6.03**	**1.74-20.84**	0.99	0.51-1.92	**2.41**	**1.11-5.24**	0.97	0.48-1.98
Swimming pool	1.26	0.76-2.08	1.66	0.72-3.83	1.04	0.54-2.03	1.24	0.45-3.40	1.41	0.68-2.91	1.18	0.40-3.49	1.14	0.59-2.21	1.84	0.66-5.12	0.83	0.35-1.95	**2.74**	**1.01-7.49**	1.57	0.60-4.07
Splash pad	**2.07**	**1.29-3.33**	**2.59**	**1.19-5.65**	1.80	0.97-3.36	2.26	0.93-5.50	1.48	0.75-2.92	**4.54**	**1.46-14.10**	**2.80**	**1.43-5.47**	1.72	0.65-4.52	2.06	0.89-4.80	**2.67**	**1.01-7.01**	2.41	0.92-6.31
Basketball court	**1.69**	**1.17-2.44**	**1.96**	**1.08-3.54**	1.49	0.92-2.42	2.00	0.89-4.52	1.51	0.91-2.50	1.94	0.85-4.45	**1.98**	**1.24-3.15**	**3.01**	**1.22-7.44**	1.34	0.65-2.77	**2.17**	**1.05-4.49**	1.52	0.75-3.11
Tennis court	**1.78**	**1.23-2.57**	**1.98**	**1.09-3.57**	**1.64**	**1.01-2.68**	2.11	0.98-4.55	1.41	0.84-2.37	**2.70**	**1.25-5.82**	**2.20**	**1.39-3.49**	2.12	0.91-4.93	1.41	0.70-2.83	1.71	0.83-3.52	1.66	0.80-3.47
Volleyball court	2.42	0.96-6.11	2.71	0.58-12.74	2.09	0.61-7.18	8.49	0.76-95.41	2.24	0.71-7.08	1.26	0.20-7.89	**3.03**	**1.12-8.17**	0.00	0.00-0.00	6.38	0.65-62.54	0.25	0.05-1.16	^2^n/a	^2^n/a
Trail	**1.65**	**1.13-2.40**	1.24	0.66-2.35	**1.88**	**1.16-3.03**	1.78	0.77-4.12	1.25	0.74-2.10	**3.17**	**1.42-7.08**	**2.07**	**1.30-3.28**	1.24	0.50-3.06	**2.08**	**1.03-4.19**	1.30	0.61-2.75	1.02	0.50-2.08
Fitness station	**7.92**	**3.05-20.57**	**18.34**	**2.47-136.18**	**5.08**	**1.61-16.07**	^2^n/a	^2^n/a	**5.66**	**1.65-19.45**	**7.92**	**1.63-38.48**	**9.88**	**3.15-30.99**	0.00	0.00-0.00	**21.34**	**2.43-187.71**	2.04	0.39-10.74	3.20	0.57-17.96
Skate park	**5.25**	**2.43-11.31**	**11.92**	**2.60-54.72**	**3.40**	**1.33-8.65**	^2^n/a	^2^n/a	**3.56**	**1.40-9.08**	**6.43**	**1.35-30.63**	**6.10**	**2.46-15.15**	0.00	0.00-0.00	**8.31**	**2.15-32.09**	1.20	0.24-5.92	3.21	0.69-14.86
Dog park	1.48	0.56-3.93	1.55	0.32-7.46	1.42	0.38-5.25	^2^n/a	^2^n/a	1.20	0.40-3.54	2.88	0.10-82.90	1.93	0.54-6.97	0.34	0.05-2.39	1.25	0.21-7.30	0.74	0.07-7.57	0.83	0.07-9.87
Green space	1.43	0.86-2.36	1.12	0.48-2.58	1.54	0.79-2.98	4.79	0.98-23.56	0.93	0.47-1.83	1.60	0.57-4.49	1.55	0.87-2.77	3.22	0.44-23.71	1.33	0.54-3.25	1.87	0.65-5.38	0.94	0.37-2.37
Lake	**1.72**	**1.15-2.58**	**2.00**	**1.05-3.82**	1.54	0.91-2.62	2.49	1.00-6.20	1.28	0.73-2.23	2.41	1.00-5.82	**1.80**	**1.11-2.92**	1.44	0.51-4.11	**2.70**	**1.20-6.08**	2.07	0.90-4.80	1.07	0.44-2.63

Bolded odds ratios and confidence intervals indicate associations that were significant at p < .05.

^1^The reference group for all park facilities analyses was those participants who did not have the specific facility within a park within 1 mile from home.

^2^n/a – Odds ratio could not be calculated because the specified facility was too uncommon within 1 mile of parks for participants in the specified demographic group.

**Table 4:** **Association of park proximity and park facilities with park-based physical activity by socio-demographic characteristics**

**Park variable**	**Full sample**		**Gender**				**Income**						**Race**				**Age**					
			**Males**		**Females**		**Low income**		**Medium income**		**High income**		**White**		**Black**		**18-39 years**		**40-59 years**		**60+ years**	
	**OR**	**95% CI**	**OR**	**95% CI**	**OR**	**95% CI**	**OR**	**95% CI**	**OR**	**95% CI**	**OR**	**95% CI**	**OR**	**95% CI**	**OR**	**95% CI**	**OR**	**95% CI**	**OR**	**95% CI**	**OR**	**95% CI**
Closest Park																						
Less than ½ mile	1.00		1.00		1.00		1.00		1.00		1.00		1.00		1.00		1.00		1.00		1.00	
½ mile to 1 mile	0.72	0.45-1.16	0.79	0.37-1.70	0.71	0.37-1.34	0.57	0.19-1.72	0.88	0.44-1.75	0.53	0.19-1.50	0.66	0.37-1.19	0.48	0.15-1.53	0.67	0.29-1.58	1.06	0.49-2.29	0.41	0.14-1.18
More than 1 mile	0.50	0.25-0.99	0.51	0.19-1.40	0.49	0.19-1.28	0.91	0.17-4.90	0.33	0.13-0.84	0.81	0.22-3.00	0.47	0.22-1.00	0.41	0.05-3.34	0.48	0.16-1.49	0.47	0.14-1.57	0.61	0.16-2.32
Number of Parks																						
0 parks	1.00		1.00		1.00		1.00		1.00		1.00		1.00		1.00		1.00		1.00		1.00	
1 park	0.97	0.46-2.04	1.45	0.48-4.38	0.73	0.26-2.04	0.29	0.04-2.30	2.62	0.93-7.40	0.25	0.06-1.08	0.88	0.37-2.04	1.41	0.15-13.02	1.12	0.30-4.20	1.07	0.31-3.66	0.69	0.18-2.68
2 parks	**2.29**	**1.06-4.95**	2.03	0.66-6.26	2.82	0.95-8.37	1.85	0.25-13.68	**3.00**	**1.04-8.67**	1.97	0.42-9.37	**2.56**	**1.10-5.97**	1.86	0.18-19.67	3.31	0.82-13.32	**3.88**	**1.08-13.98**	0.61	0.10-3.74
3 or more parks	**2.53**	**1.21-5.31**	2.85	0.93-8.80	2.69	0.95-7.63	1.02	0.15-6.97	**3.39**	**1.22-9.41**	3.25	0.64-16.49	**3.07**	**1.30-7.26**	2.49	0.28-22.43	3.02	0.9-10.12	**3.75**	**1.06-13.20**	1.28	0.29-5.59
Park Space																						
0 acres	1.00		1.00		1.00		1.00		1.00		1.00		1.00		1.00		1.00		1.00		1.00	
0.1-19.9 acres	0.96	0.47-1.98	1.02	0.35-2.97	0.93	0.34-2.52	0.15	0.01-1.56	2.38	0.88-6.45	0.34	0.08-1.40	0.91	0.40-2.05	0.96	0.10-9.46	0.95	0.25-3.51	1.15	0.33-4.03	0.61	0.16-2.43
20-49.9 acres	**2.15**	**1.03-4.48**	2.31	0.79-6.78	2.10	0.74-5.96	0.78	0.12-5.26	**3.48**	**1.26-9.41**	1.30	0.28-5.96	**2.82**	**1.21-6.57**	1.97	0.20-19.85	2.98	0.85-10.51	2.15	0.62-7.40	1.69	0.36-7.91
50 or more acres	**2.13**	**1.03-4.42**	1.49	0.50-4.47	**3.00**	**1.06-8.52**	1.66	0.32-8.62	2.58	0.94-7.12	1.93	0.38-9.96	1.84	0.78-4.34	3.39	0.36-32.16	1.73	0.51-5.83	**4.87**	**1.32-17.98**	0.65	0.13-3.30
Park Facilities^1^																						
Playground	**2.34**	**1.41-3.90**	1.53	0.71-3.29	**3.43**	**1.63-6.88**	1.74	0.43-7.05	**2.41**	**1.21-4.80**	2.22	0.76-6.49	**2.45**	**1.33-4.51**	3.27	0.83-12.85	**2.37**	**1.13-4.97**	**2.01**	**1.05-3.86**	1.48	0.68-3.23
Sports Field	1.32	0.79-2.20	1.12	0.50-2.54	1.47	0.73-2.97	0.62	0.20-1.88	**2.38**	**1.15-4.91**	0.67	0.16-2.89	1.52	0.77-3.00	1.00	0.27-3.72	1.37	0.61-3.07	1.15	0.60-2.20	0.64	0.26-1.61
Baseball Field	1.36	0.86-2.15	0.97	0.47-2.01	1.77	0.92-3.41	1.19	0.31-4.55	1.13	0.62-2.07	2.10	0.77-5.74	1.37	0.79-2.38	1.88	0.48-7.38	0.99	0.51-1.92	**3.93**	**2.10-7.33**	0.97	0.48-1.98
Swimming Pool	1.77	1.00-3.16	1.87	0.77-4.53	1.74	0.79-3.86	0.93	0.27-3.20	2.05	0.86-4.88	**4.88**	**1.11-21.51**	1.68	0.79-3.58	2.89	0.73-11.54	0.83	0.35-1.95	2.08	0.82-5.26	1.57	0.60-4.07
Splash Pad	**2.01**	**1.18-3.42**	1.88	0.74-4.80	**2.34**	**1.16-4.70**	0.97	0.32-2.96	1.63	0.73-3.63	**23.77**	**1.99-283.52**	**3.07**	**1.50-6.26**	1.46	0.47-4.54	2.06	0.89-4.80	1.98	0.88-4.44	2.41	0.92-6.31
Basketball Court	**1.97**	**1.24-3.14**	**2.43**	**1.18-5.01**	1.71	0.90-3.24	1.13	0.39-3.34	**1.91**	**1.01-3.62**	**3.12**	**1.02-9.54**	1.63	0.92-2.88	**5.25**	**1.42-19.42**	1.34	0.65-2.77	**2.31**	**1.30-4.13**	1.52	0.75-3.11
Tennis Court	**1.61**	**1.02-2.54**	1.56	0.77-3.17	1.71	0.91-3.23	0.90	0.32-2.53	1.65	0.86-3.17	2.20	0.85-5.71	1.56	0.90-2.72	2.56	0.79-8.28	1.41	0.70-2.83	**2.34**	**1.29-4.22**	1.66	0.80-3.47
Volleyball Court	0.42	0.13-1.31	0.62	0.15-2.44	0.21	0.02-1.74	0.00	0.00-0.00	0.68	0.17-2.76	0.19	0.02-1.79	0.48	0.14-1.64	^2^n/a	^2^n/a	6.38	0.65-62.54	0.70	0.23-2.15	^2^n/a	^2^n/a
Trail	1.40	0.88-2.22	0.89	0.44-1.80	**1.99**	**1.02-3.86**	0.59	0.19-1.82	**1.90**	**1.01-3.57**	1.13	0.44-2.90	1.64	0.93-2.90	1.11	0.31-3.97	**2.08**	**1.03-4.19**	**2.16**	**1.15-4.07**	1.02	0.50-2.08
Fitness Station	**3.56**	**1.50-8.45**	2.87	0.82-10.07	**5.20**	**1.57-17.22**	2.69	0.44-16.44	**5.51**	**1.16-26.23**	4.67	0.99-22.05	**6.22**	**2.02-19.17**	^2^n/a	^2^n/a	**21.34**	**2.43-187.71**	^2^n/a	^2^n/a	3.20	0.57-17.96
Skate Park	**2.47**	**1.15-5.31**	2.35	0.69-7.98	2.74	0.97-7.71	4.24	0.66-27.14	2.35	0.75-7.39	2.50	0.52-12.07	**3.73**	**1.37-10.15**	6.19	0.51-76.00	**8.31**	**2.15-32.09**	6.75	0.72-63.39	3.21	0.69-14.86
Dog Park	0.64	0.19-2.19	0.71	0.08-6.48	0.55	0.11-2.69	^2^n/a	^2^n/a	0.46	0.10-2.14	0.32	0.02-4.92	1.17	0.24-5.85	^2^n/a	^2^n/a	1.25	0.21-7.30	1.91	0.30-12.19	0.83	0.07-9.87
Green Space	1.59	0.84-3.01	1.31	0.53-3.25	1.89	0.76-4.68	1.39	0.20-9.78	2.35	0.98-5.62	0.79	0.22-2.82	1.57	0.77-3.21	1.84	0.32-10.76	1.33	0.54-3.25	2.14	0.91-5.08	0.94	0.37-2.37
Lake	1.60	0.97-2.65	1.48	0.68-3.24	1.78	0.89-3.58	2.84	0.79-10.13	1.23	0.64-2.38	1.83	0.58-5.84	1.83	0.98-3.40	0.98	0.31-3.10	**2.70**	**1.20-6.08**	1.79	0.92-3.47	1.07	0.44-2.63

Bolded odds ratios and confidence intervals indicate associations that were significant at p < .05.

^1^The reference group for all park facilities analyses was those participants who did not have the specific facility within a park within 1 mile from home.

^2^n/a – Odds ratio could not be calculated because the specified facility was too uncommon within 1 mile of parks for participants in the specified demographic group.

Compared to those with no parks, park-based PA was more likely for several groups having two and three or more parks within 1 mile, including the full sample, medium income participants, White participants, and middle-aged (40–59 years) adults (Table 4). And again, park-based PA appeared more likely as the number of parks within 1 mile of participants increased. Additionally, the full sample, medium income, and White participants were more likely to engage in park-based PA given access to 20–49 acres of park space within 1 mile. The same was true for the full sample, females, and middle-aged adults who had 50 or more acres near home (Table 4).

### Association of park facilities with park use and park-based PA

There were also positive and significant relationships with park use (Table 3) and park-based PA (Table 4) for having several specific park facilities within 1 mile from home. For example, all facilities except sports fields, swimming pools, volleyball courts, dog parks, and green spaces were associated with greater odds of park use among the full sample of participants (Table 3). Likewise, park use was related to access to splash pads, basketball courts, and lakes among males; playgrounds, baseball fields, and trails among females; and tennis courts, fitness stations, and skate parks among both genders. There were only two facilities significantly associated with park use for low income (playgrounds and baseball fields) and medium income (fitness stations and skate parks) participants, but many more among high income participants (playgrounds, baseball fields, splash pads, tennis courts, trails, fitness stations, and skate parks). All but four facilities were positively associated with park use among White participants. In contrast, only three facilities were significantly related to park use among Black participants (playgrounds, baseball fields, and basketball courts), though it should be noted that all of these three were also significant among White adults. Finally, five facilities were associated with park use among younger (18–39 years) adults, including playgrounds, trails, fitness stations, skate parks, and lakes, while four other facilities were associated with park use among middle-aged (40–59 years) adults (baseball fields, swimming pools, splash pads, and basketball courts). In contrast, none of the 14 facilities were associated with park use among older adults (60+ years) (Table 3).

Somewhat fewer statistically significant relationships were observed between the various park facilities and park-based PA (Table 4). Playgrounds were related to park-based PA among the full sample, females, medium income participants, White participants, and younger and middle-aged adults. However, sports fields, baseball fields, swimming pools, and lakes were only related to park-based PA for one sub-sample each (medium income, middle-aged, high income, and younger participants, respectively). Having a splash pad within 1 mile was positively related to park-based PA for four groups (full sample, females, high income, and White participants), while the same was true for basketball courts among six groups (full sample, males, medium income, high income, Black, and middle-aged adults). Tennis courts were important for the full sample and for middle-aged adults, while trails were related to park-based PA among females, medium income participants, and younger and middle-aged adults. Fitness stations were associated with park-based PA for several groups, including the full sample, females, medium income, White, and younger adults, while skate parks were associated with park-based PA for the full sample, White participants, and younger adults. Finally, three facilities – volleyball courts, dog parks, and green spaces – were not related to park-based PA for any of the participant samples examined.

## Discussion

Parks are important elements of the built environment as destinations and settings for PA as well as resources that can enhance the overall PA-promoting environment within neighborhoods [13,15,57]. The current study examined how multiple measures of proximity to parks and specific park facilities were associated with adults’ park use and park-based PA, and how these associations varied across several socio-demographic factors. Across all sub-samples, distance to the closest park was not significantly related to either park use or park-based PA, but numerous significant associations were found for the relationship of number of parks and amount of park space within 1 mile with both outcomes. As well, diverse facilities were associated with park use and park-based PA. For both park proximity and facilities, the significant relationships varied widely across gender, age, race, and income groups. These findings are discussed below.

### Proximity to parks

Across all sub-samples in this study, distance to the closest park was not significantly associated with either park use or park-based PA. This is consistent with some previous research which reported a lack of a relationship between access to one’s closest park and PA, although most of these studies did not use context-specific PA measures (e.g., park-based PA) [21,23,58]. For example, in a national study, Witten et al. [33] found that neighborhood access to parks (measured as travel time) was not associated with self-reported overall PA. However, there is some existing literature that emphasises the role of distance to parks on PA [17,20,26]. For instance, in a review of qualitative research, McCormack et al. [59] identified proximity as one of the important factors for encouraging park use. There are several possible reasons for this inconsistency in previous results about the effects of distance to the closest park on park-based PA. First, there may be a threshold above which the distance to the closest park affects behaviors such as walking [16]. For example, people may not walk more than 800 m (or ½ mile) to reach a park; if so, 800 m can be used as a threshold in locating a park to encourage PA. Identifying these thresholds is an important step in designing walking-friendly neighborhoods [60]. Second, diverse objective and perceived measures of proximity to parks have been used in previous studies and this is exacerbated by the fact that poor agreement often exists between these two types of measures [61,62]. Lastly, the majority of previous studies examining the influence of distance to parks on PA did not control for the attributes of the park environment (for exceptions see, [23,24]), while several previous studies showed the importance of park quality and facilities on PA [20,35]. For example, it is likely that people do not visit a park with unattractive qualities, regardless of its distance.

Nevertheless, two other measures of proximity, number of parks and amount of park space within 1 mile, were associated with measures of both park use and park-based PA for a large number of the demographic groups we examined. This finding is similar to the previous research highlighting the importance of park area in encouraging PA [16,20,25,26]. For example, Kaczynski et al. [16] found that adding each additional hectare of park area within 1 km from home was associated with increased odds of neighborhood-based moderate-to-strenuous PA. Similarly, another study found the size of urban green spaces was associated with the amount of PA related to such places [25]. These findings demonstrate the importance of park area measures (such as number of parks and park acreage) in enhancing park-based PA rather than simple measure of distance to the closest park. A greater amount of park area likely offers more diverse opportunities for residents’ park-based PA through more features in those parks or by having different types of parks. Likewise, having several parks across the neighborhood as well as other green infrastructure (e.g., greenways, trails) may create a habituation toward park-based PA by creating a neighborhood that values parks and active lifestyles. Future studies should explore the specific pathways through which park areas within a neighborhood may influence park use and park-based PA across different populations.

As mentioned earlier, distance to the closest park was not a significant factor for any of the socio-demographic subgroups. Likewise, relatively little variation in park use was observed across socio-demographic segments when considering the existence of 1 or 2 parks within 1 mile. However, when there were 3 or more parks within 1 mile of home, certain groups were more likely to report using parks within the past month. This included females and other research has shown that other active living infrastructure (e.g., bike lanes) can be particularly salient for women [63]. Likewise, low-income participants with 20 or more acres within 1 mile from home were also more likely to report using parks. Previous research has shown that neighbourhood socio-economic context plays a role in the relationship between neighborhood walkability and BMI [64] and other environmental features such as parks may be similarly important for health behaviors and outcomes in lower-income areas [65].

Compared to park use, there appeared to be somewhat less diversity among socio-demographic groups when considering associations between the number of parks, park space, and park-based PA. This was especially evident in terms of income, race, and age groups. For instance, medium income, White, and middle-aged participants were the only cohorts with increased odds of engaging in park-based PA when there were 2 parks or 3 or more parks within 1 mile. Likewise, these same three groups were the only cohorts that experienced increased odds of engaging in park-based PA when there was a greater amount of park space (20–49.9 or 50+ acres) nearby. These findings about race and income are consistent with previous research that showed Black and lower-income residents were less likely to engage in park-based PA [66], and might be explained by other studies which have documented that parks in minority and low income neighborhoods are often less attractive and appealing for PA [67,68].

### Park facilities

In addition to proximity concerns, in this study, several specific park facilities were found to be associated with both park use and park-based PA. This is consistent with an emerging body of research showing the importance of park features on PA [20,23,25,69,70]. For example, Baran et al. [70] found several park features such as playgrounds, basketball courts, pools and water features, shelters, and picnic areas to be associated with park usage among youth and adults. In another study that used a natural experiment approach, Veitch et al. [69] reported that improving the features of a park, including the establishment of a fenced leash-free area for dogs and a barbecue area, resulted in increased park use and park-based PA. It is likely that some park features are more important than others and at least one study reported that park facilities – those areas designed for active use – were somewhat more important than park amenities – features that supported PA in the park [23]. Better understanding the influence of specific park facilities on park use and park-based PA has important implications for urban designers and park planners in creating activity-friendly neighborhoods. Moreover, using certain park facilities presumably requires residents and park visitors to possess certain equipment, abilities, and other resources (e.g., time, money). Therefore, exploring how the influence of park facilities may differ across population segments is imperative.

This study advances the literature on park facilities and health behaviors by focusing on adults, by examining a wide diversity of demographic sub-groups, and by considering both park use and park-based PA. While several studies have explored park facilities that promote youth PA [36,38,40,71], it is likely that different facilities appeal to adults [35]. Park planners must work to understand the discrepancies and synergies between the preferences of these two groups and how they might be reconciled to design parks that either provide separate or integrated spaces for different generations. For example, several park facilities, including playgrounds, trails, baseball fields, and basketball courts, among others, were associated with park use and/or park-based PA within the two younger adult age groups (18–39 and 40–59 years) who presumably are more likely to have active children or be active themselves, whereas none of the 14 facilities examined here were related to park use or PA among older adults (60+ years). Several studies have shown that neighborhood resources are particularly important for older adults’ physical activity behavior [72-74], and at least one study reported that park-based leisure experiences are particularly beneficial to older adults’ physiological health [75]. Therefore, more research is needed to understand, for example, the ideal components of parks for attracting and activating older visitors.

Our study likewise revealed a wide range of park facilities that were significantly associated with park use and park-based PA for other gender, income, and race sub-populations.

The facilities most commonly associated with park use across various demographics were playgrounds, baseball fields, fitness stations, and skateparks. Therefore, these facilties may be particularly important for promoting park use for a wide range of visitor groups. Similarly, the facilities most commonly associated with park-based PA across different demographic groups were basketball courts, playgrounds, trails, and fitness stations. These findings correspond with previous research demonstrating the importance of facilities such as trails [23,35,76], basketball courts [35] and fitness zones [77], and suggest that these facilities may be especially useful in efforts to increase park-based PA among a variety of park users.

Another interesting finding was that only two facilities were significantly associated with park use for low income (playgrounds and baseball fields) and medium income (fitness stations and dog parks) participants, but many more were associated with park use among high income participants (playgrounds, baseball fields, splash pads, tennis courts, trails, fitness stations, and skate parks). Likewise, there were no facilities associated with park-based PA for low-income participants, while five facilities were signficant among medium-income (playground, sports field, basketball court, trail, and fitness station) and three for high-income participants (swimming pool, splashpad, and basketball court). Moreover, only four facilities were *not* positively associated with park use among White participants, whereas only three facilities were significantly related to park use among Black participants (playgrounds, baseball fields, and basketball courts). Similarly, four facilities were positively associated with park-based PA among White participants (playgrounds, splash pad, fitness station, and skate park), yet only one facility was related to park-based PA among Black participants (basketball court). Indeed, previous research has observed differences in park use and park-based PA based on neighborhood income and racial/ethnic composition [78]. Future research would benefit from further probing these differences to identify constraints and motivations associated with using particular facilities, especially among socio-demographic groups traditionally at risk for lower levels of park use, PA, and health.

Although few studies have considered how living close to specific park facilities is related to adults’ park use and PA [27,35], some research has reported that adult PA levels vary across park activity areas and that significant variations in within-park PA exist by gender and race [41,78,79]. Identifying specific facilities that appeal to the broadest range of users can aid park planning strategies designed to maximize the use of finite space to attract the largest number of people possible. At the same time, park planners must be conscious of issues related to conflict and crowding in considering the optimal combination of facilities and users to coexist and be active in the park. Interestingly, much research has focused on such user management issues in national parks and other large recreation areas [80,81], but not within local or municipal parks where space and activities may be even more constrained. Conversely, rather than trying to appeal to everyone, a park could be designed to target the specific population segments who live nearby, groups who may be most at-risk for psychological or physiological health issues, or other residents who might reap the greatest benefits from the inexpensive and proximal opportunities afforded by parks. For example, in this study, playgrounds were one of the key facilities associated with park use among low income participants and may be particularly important community resources for attracting families to parks for social or other health-promoting purposes [37,82,83]. In general, a wide variety of park facilities can foster park use and park-based PA among diverse groups of residents, but a better understanding of these relationships is needed to advance park design and community health for all.

### Limitations

Our study was subject to several limitations. For example, our park use and PA variables, while based on well-tested measures, were self-reported and therefore potentially subject to recall error [84]. As well, like other cross-sectional studies, we are unable to postulate as to the order of influence among the park variables and related behaviors (i.e., self-selection); consequently, more longitudinal studies of park access and features are needed across the lifespan. However, in a recent study, it was found that such self-selection issues were not problematic in that even among people with lower preferences for living near green space, those with a greater amount of proximal parkland were more likely to use parks for PA [85]. Another potential limitation was that we did not consider participants’ perceptions of access to nearby parks or the facilities therein; future research should include both objective and perceived measures of proximity to parks, which often share little correspondence [62,86], and should explore the relationships of both with different health outcomes. Additionally, although one mile is a reasonable distance within which to examine park availability and facilities among adults, using other sizes of buffers may produce different results. Likewise, using distance measures other than to the centroid of study parks may have resulted in different indicators of park proximity. In part due to space limitations, we also did not examine an exhaustive list of demographic variables (e.g., adults with children) or outcomes (e.g., meeting overall physical activity recommendations), and for certain characteristics such as race, our categories were limited by the makeup of the study respondents (e.g., primarily White and Black in this study). Finally, this study had a somewhat low response rate and focused exclusively on adults, so future research should examine these relationships among other age groups and at-risk populations (e.g., children, older adults, persons with disabilities) using population-representative samples.

## Conclusions

The current study contributes to the limited, but fast-growing and important body of literature examining the influence of park attributes on adults’ PA. Other research has explored effects of park proximity or specific facilities across diverse demographics among youth [40,87,88], but this is one of the first such studies to consider these issues in detail among adults. We found that specific measures of park proximity – namely, the number of parks and total park area within 1 mile from home – as well as a wide variety of park facilities were associated with both park use and park-based PA among diverse gender, income, race, and age groups. Given this complexity, future efforts to understand and design health-promoting parks may benefit from interdisciplinary teams involving researchers and professionals from fields such as parks and recreation management, leisure studies, landscape architecture, geography, urban planning, psychology, sociology, and public health, among others. Overall, findings from this and similar ongoing studies should be considered in better designing activity-friendly parks and communities that are demographically- and culturally-responsive to the needs of neighborhood residents.
